# Understanding of guest behavioral intentions in peer-to-peer accommodation sector

**DOI:** 10.3389/fpsyg.2022.1008226

**Published:** 2022-10-20

**Authors:** Ye Ye, Laiba Ali, Foong Yee Wong, Siew Imm Ng, Xin-Jean Lim

**Affiliations:** ^1^Business School, Shaoguan University, Shaoguan, China; ^2^School of Economics and Management, South China Agricultural University, Guangzhou, China; ^3^School of Business and Economics, Universiti Putra Malaysia, Serdang, Malaysia; ^4^Faculty of Economics and Management, Universiti Kebangsaan Malaysia, Bangi, Malaysia

**Keywords:** behavioral intentions, guest emotions, guest satisfaction, peer-to-peer accommodation, physical environment, social environment

## Abstract

The purpose of this study is to investigate the antecedents of guests’ behavioral intentions in Malaysia’s peer-to-peer (P2P) accommodation industry. This study focused on the effects of physical and social environment on guest emotions, satisfaction, and subsequently on guest’s behavioral intentions towards P2P accommodation. The proposed research framework was developed based on Stimulus-Organism-Response model. Partial Least Square Structural Equation Modeling (PLS-SEM) was used to examine the proposed hypotheses. Data were collected from 476 foreign visitors who stayed at P2P accommodations in Malaysia using online survey. The findings demonstrated that the P2P accommodation’s social and physical environment had a positive impact on guest satisfaction and emotions, while both satisfaction and emotions had significant impact on guest behavioral intentions. The findings also extend the applicability of the S-O-R theory in P2P accommodation context. In practice, both the physical and social environments are important stimuli cues to induce favorable level of organism factors, i.e., satisfaction and emotions. While behavioral intentions acted as the response factor in examining visitor’ responses towards P2P accommodation. All in all, this study emphasizes how crucial it is to emphasize on the external and internal factors when encouraging positive response in P2P accommodation platforms.

## Introduction

Understanding the customers’ purchase intentions and predicting their future purchasing intentions was a critical responsibility for business managers. Recently, the sharing economy and especially the peer-to-peer (P2P) accommodation is becoming more popular and shave disturbed the conventional accommodations sector (Yan et al., 2019; Kuhzady et al., 2020). The sharing economy is a socio-economic ecosystem model based on sharing, renting, swapping, lending, exchanging, collective purchasing, co-creation and borrowing. In details, it refers to how common people would exchange resources or properties for a fee over the internet or other mobile technologies (Kuhzady et al., 2020). According to Belk (2014), the P2P is considering a subset of sharing economy, as it mainly focuses on short-term lodging businesses.

This industry’s appeal as lodging option and had have contributed to ongoing innovation in the lodging and tourist sectors (Guttentag et al., 2018). The major players in the P2P accommodation sector include Homestays, Airbnb, Homeaway, Wimdu, Roomorama, and 9flats (Tussyadiah, 2016; Guttentag et al., 2018). Among them, Airbnb is the main player in P2P lodging and is gaining ground. Despite being a relative newcomer to other hotels in the hospitality sector, Airbnb has emerged as a formidable rival (Tussyadiah, 2016). Due to its widespread use in the P2P lodging market, Airbnb is regarded as the most effective tool (Liu and Mattila, 2017). The Airbnb innovation has completely changed the lodging business and could potentially have an impact on the hotel industry (Nawi et al., 2019).

In 2015, P2P lodging, particularly Airbnb, attracted a lot of interest in Malaysia (Euromonitor International, 2016). Malaysia has welcomed visitors from 78 different nations, and it was rated third in terms of bookings, behind Thailand and Japan (Razli et al., 2017). The popularity of Malaysia’s homestay program is also rising daily among housing options (Balasingam et al., 2017). These were divided into 193 clusters, which together with 3,800 homestay providers brought in MYR29.66 million (a USD1.00 is equivalent to MYR4.54) in visitor revenue in 2019. However, due to COVID-19 pandemic, it has dropped to MYR6.13 million in visitor revenue in 2021 (Ministry of Tourism and Culture, 2022). More specifically, the accommodation industry has made it possible to improve the economic standing of the entire rural population. When compared to regular accommodations, most tourists now favor P2P accommodations (Guttentag et al., 2018). Due to the importance of this industry, it is necessary to identify the antecedents that contribute to guests’ behavioral intentions when staying in P2P accommodations.

Numerous studies have discovered that emotions and satisfaction are the key factors influencing customers’ behavioral intentions regarding resort hotels and other hospitality sectors (Sherman et al., 1997; Chen et al., 2013; Jani and Han, 2013; Prayag et al., 2013; Ali and Amin, 2014). Similar to this, studies in the field of services marketing have concentrated on discovering the elements that could raise customer satisfaction and emotions. According to previous studies, the social and physical environment has a significant impact on customers’ emotion (Sherman et al., 1997; Ryu and Jang, 2007; Kim and Moon, 2009; Pelegrin-Borondo et al., 2017) and satisfaction (Ryu and Jang, 2007; Kim and Choi, 2013; Ali and Omar, 2014; Cetin and Dincer, 2014). The way that guests are treated is greatly influenced by their emotions. They tracked guests’ emotions in real time throughout their journey and discovered that they fluctuate as they visit places with varying levels of experience and interest (Tuerlan et al., 2021). Additionally, P2P accommodations provider greater satisfaction in addressing a variety of tourist needs, such as the need for more social needs, authentic experiences, sustainable travel, etc. (Tussyadiah, 2016). As a result, distinct influencing elements may be connected between guests staying in affiliated hotels and those who use P2P accommodations.

Service providers of P2P accommodations must first take advantage of the distinctive service delivery to sustain growth. Given that customers have few ways to gauge the quality of the services, focusing on the physical environment has shown to be an effective way to get a competitive edge (Ali and Amin, 2014). The physical environment has been regarded in the hospitality sector as a crucial tangible cue to test services (Bitner, 1992; Hightower and Shariat, 2009). Layout, accessibility, facility aesthetics, functionality, and cleanliness are physical environment factors that have a substantial impact on the pleasure and arousal aspects of air travelers’ emotions in the context of air travel (Tuerlan et al., 2021). Therefore, service providers invest a lot of money in creating attractive physical spaces.

The development of the hotel sector has helped people become more independent thanks to technological advancements and given them greater freedom to travel as they see fit. Therefore, personal participation cannot be found in modern hospitality (Wood, 1994). People no longer have time to spare for travel or even simple social interaction. As compared to other accommodation sectors, P2P accommodation meets the tourists’ diverse social needs by allowing them to interact with new people (locals) and have real experiences (Tussyadiah, 2016). As a result, it can be claimed that people on vacation are unwilling to make compromises regarding the social or physical environments. Providing excellent services with the aid of the social and physical environment can boost visitor satisfaction and future intentions (Ali et al., 2016). Additionally, as part of professional services like P2P accommodation, the environment (social and physical) is continuously created over time to heighten guests’ pleasure and other emotions (Wakefield and Blodgett, 1999). However, academics advocate evaluating the significance of emotions in the hospitality industry to comprehend how the physical and social environment affects behavioral intentions as opposed to hotels (Ali and Omar, 2014). The research also suggests analyzing different fundamental responses from guests while being aware of how the social and physical environment affects behavioral intentions (Han and Ryu, 2009; Ali and Amin, 2014; Ali and Omar, 2014; Ali et al., 2016). Considering the aforementioned gaps, this study aims to assess the impact of the social and physical environments, as well as the guests’ satisfaction and emotions, on their behavioral intentions related to their stay in a P2P accommodation.

## Literature review

### Stimulus-organism-response model

The stimulus-organism-response model (S-O-R) was developed by Mehrabian and Russell in 1974. S-O-R model comprising of stimulus, organism and response. It is an extension to the classic theory of the stimulus–response model suggested by Pavlov in 1927 (Pandita et al., 2021). It holds that, the basis for decision-making is people’s internal emotions or sentiments, referred to as organisms, which are influenced by external stimuli like the environment and can alter behavioral intentions. Specifically, “stimulus” symbolizes various environmental and other inducements (Jang and Namkung, 2009; Ali et al., 2016); “organism” is made up of inner processes (such as emotions, perceptions, and satisfaction) that are a result of a person’s responses and actions. This suggests that exterior inputs, as well as initial emotional and internal states, directly influence approach and avoidance responses in environmental scenes (Rose et al., 2012). Overview of literature demonstrated that the S-O-R model has been used to understand consumer behavior in retail and service sectors, including the environment in stores and malls, trade marketing, resort hotels, and restaurants (Baker et al., 1994; Wakefield and Baker, 1998; Kaltcheva and Weitz, 2006; Jang and Namkung, 2009; Ali et al., 2016). All these research findings suggested that the environment has an impact on customers’ emotions and satisfaction, which in turn affects customers’ behavioral intentions.

According to the stimulus-organism-response theory, the researcher in this study investigated how stimuli and an organism affected guests’ behavioral intentions. The primary focus was on the guests’ behavioral intention, and the determinants of behavioral intention, including the physical environment, social environment, guest emotions, and satisfaction, were examined. The physical environment and social environment were included in the stimulus since they have an external influence on people’s behaviors. Three other dimensions made up the physical environment: the ambient component, the design component, and the spatial layout. In contrast, the organism also contained guest emotions and guest satisfaction, which are thought of as personal internal states of individuals. In order to determine whether or not P2P accommodations will be approached or avoided by guests, this study looked at how the physical and social environments impact their internal states (emotions and satisfaction).

### Physical environment

P2P accommodations address the demand for visually appealing and distinctive physical spaces (Han and Ryu, 2009; Pareigis et al., 2011; Ali et al., 2016; Tuerlan et al., 2021). In this situation, the physical environment is crucial in influencing the experiences of guests and differentiating service providers. Therefore, the physical environment may be seen as an external appearance that enables the service provider to boost customer expectations by providing marks in terms of value intangible services *via* tangible indications (Berry and Parasuraman, 2004; Ali et al., 2016). Wakefield and Blodgett (1994) also disputed the idea that there is a greater likelihood that the physical environment will have an impact on consumers’ behavioral intentions when it comes to leisure amenities where guests place a higher priority on relaxing.

There were some differences across service organizations’ integrated physical environment dimensions, according to academic debate on several physical environment dimensions (Ali et al., 2016). Bitner (1992) explained physical environment as the “servicescape” and classified it into three dimensions: (1) the ambient environment; (2) the spatial layout and functionality; and (3) signs, symbols, and artefacts. Ambient components, also known as ambient conditions, were thought to be the intangible physiological aspects of the environment, such as sound, cleanliness, temperature, and smell. Information about service facilities is provided by tangible aspects of the environment, such as symbols, signs, artefacts, and design elements. Last but not least, size, arrangement, and shape of the furniture, machinery, or equipment that allows addressing the needs of the guests are referred to as functionality and spatial layout (Ali et al., 2016; Moon et al., 2016). Other researchers proposed crucial parameters for the physical environment in many industries based on these dimensions, as shown in Table 1. Despite the fact that there have been many earlier studies on how physical environments may affect various businesses, there have been very few studies on the physical environment as it relates to P2P accommodation. An understudied subject is the multidimensional impact of physical environment pertaining to P2P accommodations on guest emotions, behavioral intentions, and satisfaction. A study conducted by Tussyadiah (2016) investigated how P2P accommodations amenities would impact guests’ future intentions and satisfaction.

**Table 1:** Physical environment dimensions.

Authors	Physical environment dimensions	Industry	Country
Bitner (1992)	1. Ambient conditions 2. Space and function 3. Spatial layout and functionality	Service organizations	Not Provided
Baker et al. (1994)	1. Ambient Factors 2. Design Factors 3. Social Factors	Retail	United States
Wakefield and Blodgett (1996)	1. Layout accessibility 2. Facility Aesthetics 3. Seating Comfort 4. Electric Equipment and Displays 5. Cleanliness	Leisure service settings	United States
Wakefield and Blodgett (1999)	1. Building Design and Décor 2. Equipment 3. Ambience	Leisure service settings	United States
Lucas (2003)	1. Layout Navigation 2. Cleanliness 3. Seating Comfort 4. Interior Decor 5. Ambient Factors	Casinos	United States
Kottasz (2006)	1. Exterior Factors 2. Interior Factors 3. Layout and Design 4. Decorations 5. Human Factors	Museums	United Kingdom
Ryu and Jang (2007)	1. Facility Aesthetics 2. Lighting 3. Ambience 4. Layout 5. Dining Equipment	Upscale restaurants	United States
Kim and Moon (2009)	1. Ambient Conditions 2. Facility Aesthetics 3. Layout 4. Electric Equipment 5. Seating Comfort	Theme restaurants	Canada
Han and Ryu (2009)	1. Decor and Artifacts 2. Spatial Layout 3. Ambient Conditions	Restaurants	United States
Han (2013)	1. Ambient Conditions 2. Space/Function	Low-cost airlines	Asian countries
Wu et al. (2018)	1. Design 2. Equipment 3. Environment 4. Ambience 5. Social Factors	Theme parks	Taiwan
Wang and Mattila (2015)	1. Physical Setting 2. Service Provider 3. Other Customers	Ethnic restaurants	United States
Moon et al. (2016)	1. Layout Accessibility 2. Facility Aesthetics 3. Functionality 4. Cleanliness	Airports	Korea
Ali et al. (2016)	1. Layout Accessibility 2. Facility Ambience and Aesthetics 3. Functionality 4. Cleanliness	Airports	Malaysia

Source: Ali et al. (2016).

### Social environment

Staying in P2P accommodation also enables customers to satisfy their social requirements, such as making new friends, networking, forming associations, learning about different cultures, and joining a community (Möhlmann, 2015). Due to sharing, there was an increase in the number of visitors to the P2P accommodation and keeping positive social ties with the P2P providers (Kim et al., 2015). The consumer’s desire to participate in online communities is realized with the aid of P2P accommodation and other organizations that promote collaborative consumption (Möhlmann, 2015; Tussyadiah, 2016). P2P accommodations allow for direct communication between the host and guest, which promotes social connections rather than business transactions (Kim et al., 2015). As a result, the social environment of a P2P accommodation affects the behavioral intentions of its guests for future use. P2P accommodation can facilitate the acquisition and distribution of underutilised real estate resources among individuals, contribute to residents’ incomes, allow guests to establish community connections with the host community, and foster cross-cultural understanding (Stienmetz et al., 2020).

The elements of the social environment have also been covered by several experts. According to Brady and Cronin (2001), the social environment is characterized by individual communication and includes the elements of behavior, attitude, and competence. As shown in Table 2, other sub-features of the social environment in various situations and countries have also been presented based on these aspects.

**Table 2:** Social environment dimensions.

Authors	Social environment dimensions	Industry	Country
Brady and Cronin (2001)	1. Attitude 2.Behavior 3.Expertise	Fast food, photograph developing, amusement parks and dry cleaning	Not provided
Caro and Garcia (2008)	1. Conduct 2. Expertise 3. Problem solving	Travel agencies	Spain
Walls et al. (2011)	1. Attitude 2. Professional behavior 3. Proactive service 4. Appearance	Luxury hotels	United States
Chen et al. (2013)	1. Employee’s expertise 2. Employee’s problem-solving skills	Bed & Breakfast market	Kinmen Island
Cetin and Dincer (2014)	1. Interaction with employees 2. Interaction with customers	Five-star hotels	Turkey
Ali and Omar (2014)	1. Interaction with staff 2. Interaction with other customers	Resort hotels	Malaysia

### Guest emotions and satisfaction

Guest emotions and satisfaction about using P2P accommodations provide a deeper understanding of the sharing economy (Tussyadiah, 2016). Certain positive as well as negative personal feelings are considered to be emotions. Numerous specialists have identified how important emotions are in determining service quality, choosing service providers, remaining loyal to a location, and returning to the same location (Martin et al., 2008; Ali et al., 2016). Additionally, Lee et al. (2009) hypothesized that positive emotions influence decision-making more quickly than negative ones. These exhibit a preference for decision-making even when they are not responses to assessments (Martin et al., 2008). The primary goal of several service sectors is to increase fulfillment since customer satisfaction has a direct impact on revenue, loyalty and behavioral intentions (Bowen and Chen, 2001; Chen et al., 2020). Consequently, emotion is a significant result of the guests experience and pleasant feelings are associated with customer satisfaction and loyalty (Chen et al., 2020). To estimate the behavioral intentions of the guests to return to the P2P accommodation on their following trips, emotions and guest satisfaction play a significant part (Tussyadiah, 2016).

### Behavioral intentions

According to Wang et al. (2021), the signal of a person’s readiness to carry out a particular behavior is reflected in their behavioral. There are linked favorable or unfavorable indicators that help determine if a customer will use a service or a flaw from the service provider (Zeithaml et al., 1996). Favorable behavior intentions include being helpful, being more loyal to the service provider, spending more on the services, spreading good word of mouth, and getting the greatest deal. Unfavorable behavioral intentions, however, include client churn, deterrent behavior, decreased spending with the service provider, no further referrals, and unfavorable word-of-mouth (Ladhari, 2009; Ali and Amin, 2014). To increase sales and revenue, it is crucial for service providers to understand the variables influencing guest behavioral intentions. This is because it encourages motivating behaviors and repeat visitations of P2P accommodations. The readiness to revisit and the willingness to refer others with relation to the post-consumption assessed judgment of P2P accommodation are considered behavioral intentions in this study, which is in accordance with the literature.

## Hypotheses development

**Figure 1:**
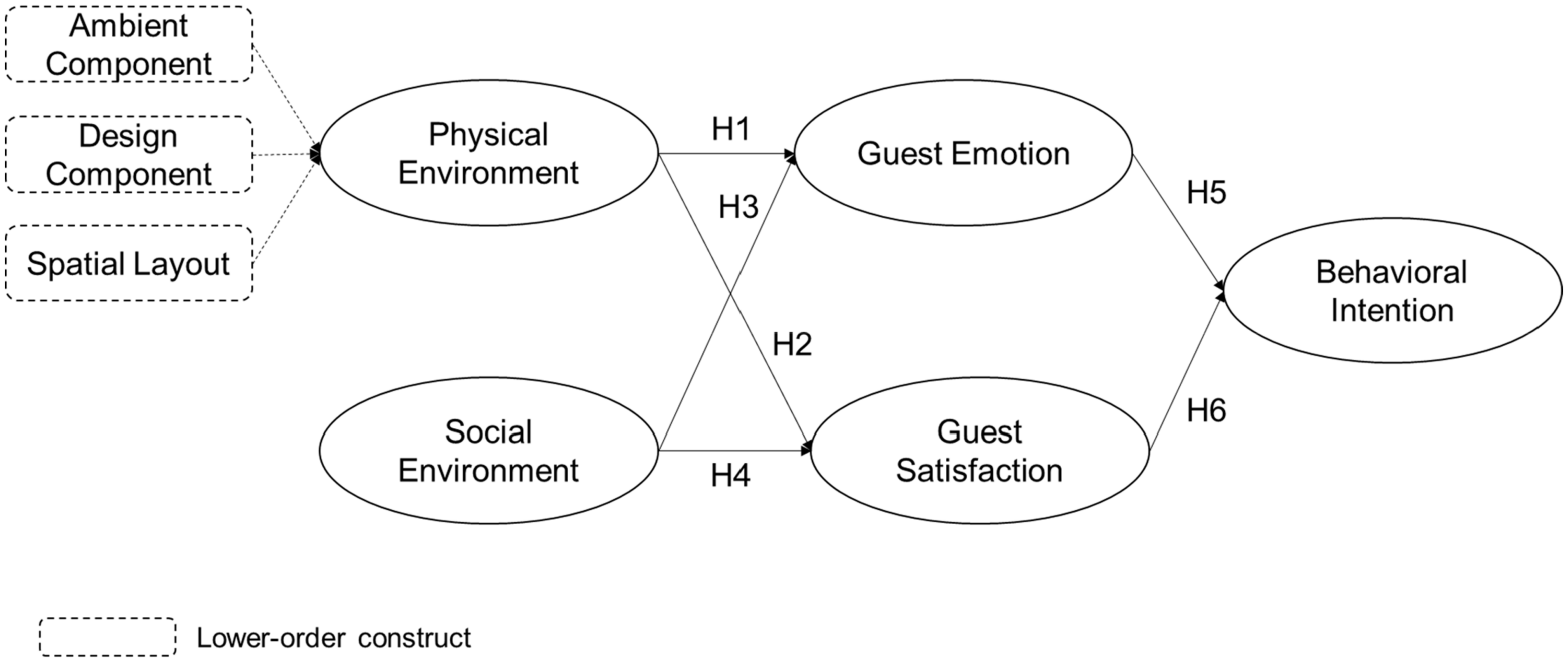
Research framework.

Based on the S-O-R (Stimulus-Organism-Response) model, this research has developed a framework for the guests’ behavioral intentions prior to their stopover at the P2P accommodation. This study proposed that physical and social environments of P2P accommodations will stimulate guest emotions and levels of satisfaction, which in turn, influence behavioral intentions. As showed in Figure 1, the social and physical environment serves as a proxy for motivation, while satisfaction and guest emotions representing by the guests’ internal organism, and their behavioral intentions serve as a proxy for reaction.

### Physical environment on guest’s emotions and satisfaction

Studies have established that visitors’ emotions, degree of satisfaction, and cognitions are influenced by their physical surroundings (Kim et al., 2009; Ladhari, 2009; Nawi et al., 2019), and they have also demonstrated empirically that visitors’ emotions alter in response to their physical environments (Bitner, 1992). According to Ladhari's (2009), customers’ emotional responses to their physical environment can alter their level of satisfaction. Physical surrounds at an accommodation location, including ambiance, design element, and environment in a service setting, including ambiance, design elements, structural layout, and sanitation, evokes emotions and influence the degree of customers satisfaction (Wakefield and Blodgett, 1996; Ali et al., 2016). Studies conducted by Nawi et al. (2019) and Sharma and Stafford (2000) found that by encouraging service businesses to attract and retain consumers, physical environments may have an impact on customers’ emotions. Ali and Amin (2014) also claimed that physical environments have a significant influence in appealing to guests’ emotions and degree of satisfaction in their research on resort accommodations. Two hypotheses were developed based on the importance of the physical environment in drawing the consumers’ emotions and degree of satisfaction:

*Hypothesis* 1. Physical environment in P2P accommodation has a positive impact on guest emotions.

*Hypothesis* 2. Physical environment in P2P accommodation has a positive impact on guest satisfaction.

### Social environment on guest’s emotions and satisfaction

The literature has extensively covered the impact of social context on emotions and level of satisfaction. Due to direct interaction, experts believe that service providers can influence visitors’ emotions, sensitivities, satisfaction levels, and behavioral intentions when they are in a service place (Mittal and Lassar, 1996; Brunner-Sperdin and Peters, 2009). The emotional state and level of satisfaction of customers are significantly influenced by interaction quality. Numerous studies have investigated the connection between customers’ interactions with others and their satisfaction and discovered that interactions with others are closely related to positive feelings (Claffey and Brady, 2019; Chen et al., 2020). Communication between guests and hosts, as well as amongst guests, has a significant impact on how they behave (Kim and Choi, 2013). Walls et al. (2011) asserted that social environment influences the degree of customer satisfaction in their study of luxurious hotels. Brunner-Sperdin et al. (2012) also highlighted that staff members’ physical appearance, hygiene, dress code, haircuts, and makeup might have a big impact on consumers’ emotions. According to Tussyadiah's (2016) evaluation, there is a significant relationship between visitors’ levels of satisfaction and their social environment. Additionally, Ali and Omar (2014) noted that social environments are reliable predictors of guests’ levels of satisfaction and behavioral intentions in Malaysian resort hotels, which led to the development of the following hypotheses:

*Hypothesis* 3. Social environment in P2P accommodation has a positive impact on guest emotions.

*Hypothesis* 4. Social environment in P2P accommodation has a positive impact on guest satisfaction.

### Guest emotions and satisfaction on behavioral intentions

Personal satisfaction triggers approach behaviors including loyalty, revisit intention, and desire to pay more. On the other hand, people’s degrees of dissatisfaction are positively correlated with avoidance behaviours including switching intentions and complaining (Tuerlan et al., 2021). Since emotion motivates people to make decisions and take action, Bergenwall (1998) referred to emotion as action oriented. In service industries, the degree of customer satisfaction is seen as a key determinant of future behavior (Taylor and Baker, 1994). According to Jang and Namkung (2009), behavioral intention should be founded on the customers’ emotions. They also anticipated that positive emotions experienced while providing services would result in more benevolent behavioral intentions. Customer satisfaction and behavioral intention are qualitatively different, according to Mittal et al. (1998). Several service industries, particularly the behavioral intention, have the goal of increasing customer satisfaction because it directly impacts revenue (Bowen and Chen, 2001). Chang (2016) has also noted that customers’ behavioral intentions and recommendations to other potential customers are outcomes that are influenced by emotions. To raise behavioral intention to revisit, it is essential to promote higher levels of satisfaction (Ali and Amin, 2014). Tussyadiah (2016) examined guest satisfaction and revisited behavioral intentions in peer-to-peer accommodation, which led to the development of the following two hypotheses:

*Hypothesis* 5. Guest emotions in P2P accommodation have a positive effect on behavioral intentions.

*Hypothesis* 6. Guest satisfaction in P2P accommodation has a positive effect on behavioral intentions.

## Methodology

### Data collection procedure

Questionnaires were distributed to P2P tourists who had stayed at the Malaysian P2P accommodations using Amazon Mechanical Turk. Out of a total of 504 replies, 476 were completed and were used for further assessment. The sample size satisfied the suggested minimum requirement based on the criteria of 99% confidence level, standard deviation 0.5, and ± 1% error margin. Table 3 lists the demographic characteristics of the respondents. Particularly, 50.4% of the respondents were male and 49.6% were female, 45% were in the 25 to 34 age range, 79.4% percent had bachelor’s degrees, the majority were from Europe (36.6%), and 31.9% said they made between $4,000 and $6,000 a year. Additionally, 69.1% of the respondents had stayed in a P2P accommodation 1–3 times in the previous two years, with 80.7% of leisure visitors and 50.2% of those staying in P2P accommodations through homestays.

**Table 3:** Demographic characteristics of the respondents.

**Demographic characteristics**		**Percentage (%)**
Gender	Male	50.4
	Female	49.6
Age	18–24	19.1
	25–34	45
	35–44	21.8
Education	School/High School	6.3
	Bachelors	79.4
Nationality	Asian	28.6
	European	36.6
	American	22.3
Income	Under $2000	12
	$2001 - $4,000	25.8
	$4,001 - $6,000	31.9
No of time stayed	1–3	69.1
	4–6	24.8
	7–9	5.7
Purpose of traveling	Leisure	80.7
	Business	13.2
	Others	6.1
P2P accommodation platform	Airbnb	43.7
	Homestay	50.2
	Others	6.1

### Measurement

The three-dimensional, ambient component, design component, and spatial outline scale for measuring physical environments was adapted from Bitner (1992). While the satisfaction measure was taken from Westbrook and Oliver (1991), the social environment scale was taken from Ali and Omar (2014). The emotion measure was adapted from Mehrabian and Russell (1974) and the scale from the investigations of Möhlmann (2015) and Tussyadiah (2016) was used to assess behavioral intentions. In this study, a five-point Likert scale with a rating of 1 (strongly disagree) to 5 (strongly agree) was utilized. Then, a pilot examination was conducted to investigate the reliability and legitimacy of the scales employed.

### Data analysis tool

To investigate the proposed relationship, the Partial Least Square Structural Equation Modeling (PLS-SEM) method was used. PLS is a well-established technique for estimating the path coefficients of structural models that has gained popularity over time. This method is undoubtedly the most suitable analytical tool because the goal of this research is to forecast the interrelationship between social and physical environments, satisfaction, and emotions on behavioral intentions (Ali et al., 2018). The provided standard criteria were used to divide the data analysis process into two stages. First is to look at the construct’s validity and reliability in the assessment of measurement model, and second is to examine the path coefficients of the hypotheses in the structural model.

## Results

### Measurement model

The assessment of measurement model includes examining internal consistency, convergent validity, and discriminant validity (Hair et al., 2006). First, the outer loading of all items was above threshold value of 0.4 (Chin et al., 2008), while the values of composite reliability (CR) were greater than 0.5. Thus, it can be concluded that internal consistency was established. Second, the average variance extracted (AVE) scores were greater than 0.5, demonstrating satisfactory convergent validity (Ali et al., 2018) (see Table 4). Last, the discriminant validity was measured using the heterotrait-monotrait (HTMT) ratio. As presented in Table 5, all constructs with HTMT less than 0.85. This therefore indicated all the constructs were distinct among each other.

**Table 4:** Results of internal consistency and convergent validity.

**Construct**	**Items**	**Loadings**	**AVE**	**CR**
Ambient component	AC1: The property has a pleasant smell.	0.801	0.705	0.923
	AC2: Lighting at the property creates a pleasant atmosphere.	0.842		
Design component	DC1: The property’s physical facilities are comfortable.	0.569	0.52	0.906
	DC2: Property’s interior layout is pleasing.	0.791		
	DC3: The signs used (i.e., bathroom, enter, exit, smoking) are helpful to me.	0.75		
	D4: The restroom(s) is/are appropriately designed.	0.737		
	D5: The property’s parking lot has more than enough space.	0.703		
	D6: The color scheme is attractive.	0.803		
	D7: The materials used inside the property are pleasing and of high quality.	0.802		
	D8: The architecture is attractive.	0.675		
	D9: The style of the interior accessories is fashionable.	0.619		
Spatial layout	SL1: Layout of the property makes it easy for me to move around.	0.866	0.745	0.897
	SL2: Furniture arrangements give me enough space.	0.891		
	SL3: Furniture arrangements make me feel comfortable.	0.831		
Social environment	SE3: Host at the property is friendly.	0.499	0.536	0.872
	SE4: Host at the property provides the service as promised.	0.795		
	SE5: Other guests at the property are well dressed and clean.	0.758		
	SE6: Other guests at the property are considerate of privacy of others.	0.804		
	SE7: Other guests at the property are friendly.	0.758		
	SE8: Other guests at the property help me, if needed.	0.733		
Guest emotions	GE1: My stay at the property makes me feel happy.	0.689	0.603	0.899
	GE2: My stay at the property makes me feel pleased.	0.871		
	GE3: My stay at the property makes me feel satisfied.	0.891		
	GE4: My stay at the property makes me feel contended.	0.814		
	GE5: My stay at the property makes me feel hopeful.	0.821		
	GE6: My stay at the property makes me feel relaxed.	0.507		
Satisfaction	SAT1: I am satisfied with my decision to stay at this property.	0.804	0.668	0.889
	SAT2: My choice to choose this property is a wise one.	0.827		
	SAT3: I think I did the right thing to stay at this property.	0.85		
	SAT4: I feel that my experience with this property is enjoyable.	0.787		
Behavioral intention	BI1: I will continue to stay at a P2P accommodation in the future.	0.862	0.81	0.927
	BI2: I will recommend my friends and family members to stay at a P2P accommodation in the future.	0.907		
	BI3: It is likely that I will use a P2P accommodation in the future.	0.929		

**Table 5:** Results of discriminant validity.

**Constructs**	**1**	**2**	**3**	**4**	**5**	**6**	**7**
Ambient components							
Behavioral intention	0.475						
Design components	0.695	0.538					
Guest emotions	0.464	0.608	0.731				
Social environment	0.421	0.575	0.679	0.716			
Spatial layout	0.553	0.524	0.821	0.710	0.688		
Guest satisfaction	0.403	0.732	0.511	0.695	0.520	0.589	

### Higher-order construct

In this study, physical environment was specified as reflective-formative higher order construct (HOC) which comprised three sub-dimensions: design components, ambient components and spatial layout. As shown in Table 6, all the three dimensions are significant regressed towards physical environment at value of p less than 0.001. Thus, it can be concluded that physical environment was formatively formed by three sub-dimensions.

**Table 6:** Results of higher-order construct.

**Higher-order construct**	**Sub-dimension**	**Weights**	***T*-value**	***P*-value**
Physical environment	(i) Ambient components	0.812	35.697	0.000
	(ii) Design components	0.941	121.471	0.000
	(iii) Spatial layout	0.804	38.874	0.000

### Structural model

The bootstrapping procedure using sub-sample of 5,000 was employed to examine the proposed hypotheses. As presented in Table 7, physical environment of P2P accommodation was found to positively impact on emotions (H1: *β* = 0. 444; *p* < 0.01) and satisfaction (H2: *β* = 0.355; *p* < 0.01). Likewise, the social environment of P2P accommodation was found positively impacted on guest emotions (H3: *β* = 0.337; *p* < 0.01) and satisfaction (H4: *β* = 0.224; *p* < 0.01). While, behavioral intentions of the guests were found to be positively impacted by guest emotions (H5: *β* = 0.239; *p* < 0.01) and guest satisfaction (H6: *β* = 0.494; *p* < 0.01). Thus, all the hypotheses, i.e., H1–H6 were supported. In terms of explanatory power, 27% of the variance of guest satisfaction and 48.70% of the variance of guest emotions were explained by physical and social environments. In addition, 43.8% of the variance of guest behavioral intentions was explained by a combination of satisfaction and emotion.

**Table 7:** : Results of path coefficient.

Hypothesis	Beta	*p*-value	95% CI	*F* square	*Q* square
H1: Physical environment → guest emotions	0.444	0.000	(0.373; 0.512)	0.252	0.277
H2: Physical environment → guest satisfaction	0.355	0.000	(0.256; 0.451)	0.113	0.171
H3: Social environment → guest emotions	0.337	0.000	(0.274; 0.402)	0.145	
H4: Social environment → guest satisfaction	0.224	0.000	(0.120; 0.328)	0.045	
H5: Guest emotions → behavioral intention	0.239	0.000	(0.157; 0.322)	0.068	0.338
H6: Guest satisfaction → behavioral intention	0.494	0.000	(0.414; 0.571)	0.289	

Subsequently, Cohen’s (1998) was used to analyze the effect sizes (*f*^2^) of all the exogenous variables. Generally, effect size of 0.02, 0.15, and 0.35 was interpreted as small, medium and large, respectively. In this study, both H1 and H6 were found demonstrated a medium effect size, while H2, H3, H4 and H5 illustrated a small effect size. Lastly, the blindfolding procedure was employed to evaluate the model’s predictive relevance. Since all the endogenous variables exhibited a Q^2^ value greater than 0 (ranged between 0.171 to 0.277), it can be concluded the present model having reasonable level of predictive relevance (Hair et al., 2012).

## Conclusion and implications

P2P accommodation has steadily gained viability as an accommodation alternative for travelers. With more locals opening their homes to guests, it is crucial to gain a deeper understanding of the elements influencing the guests’ behavioral intentions. The effect of social environment on the emotions and behavioral characteristics of the guests has been disregarded for a long time, even though there are numerous research studies on the impact of physical environment on behavioral intention and these have attracted the attention of various researchers. The guests’ interactions with the social and physical environment shape their outcomes, including their behaviors and emotions, based on their experiences. However, few studies have focused on the function of satisfaction and emotions and measured the impact of the social and physical environments on the customers’ behavioral responses (Ali and Amin, 2014; Ali and Omar, 2014; Tuerlan et al., 2021). Numerous researchers have emphasized the importance of examining the significance and effects of social environment, physical environment, and individual internal states on revisit intentions of the guest based on their stay at P2P accommodations (Guttentag et al., 2018; Claffey and Brady, 2019; Chen et al., 2020).

This study intends to identify the relationships between the social and physical environments of a P2P accommodation and the satisfaction, emotions, and behavioral intentions of its guests. In relation to the goals and objectives of the study, six hypotheses were tested. The review of literature indicated various antecedents of behavioral intentions which include the physical environment, social environment, emotions, and satisfaction. In this study, the S-O-R theory was used to evaluate how these antecedents affected the behavioral intentions of guests while they were staying in P2P accommodations. The findings, which were based on structural equation modeling, were found to support all six hypotheses. The results showed that the physical environment of the P2P accommodation would impact the guest’s emotions and satisfaction, which served as the strongest stimuli that may affect the guest’s behavioral intentions. This shows that the design, layout, and facilities associated to P2P accommodation boosted the customers’ satisfaction, emotions, and behavioral intentions.

In this study, 43.8% of the variance in the guest behavioral intentions was explained by the predictors. In addition, the good behavior and appearance of the hosts or other guests who were staying in Malaysia’s P2P accommodations tended to delight, please, and satisfy the guests. When there were higher levels of pleasant emotions and satisfaction, it led to repeat visits and behavioral intentions toward using P2P accommodations. The study’s conclusions aligned with other works on the sharing economy and services marketing (Bitner, 1992; Wakefield and Blodgett, 1996; Jang and Namkung, 2009; Ladhari, 2009; Walls et al., 2011; Brunner-Sperdin et al., 2012; Ali and Omar, 2014; Ali et al., 2016; Tussyadiah, 2016; Kuhzady et al., 2020).

This study places a strong emphasis on the factors influencing the guest’s behavioral intentions throughout their stay at a P2P accommodation, which could add to the body of knowledge already available on hospitality management. The behavioral intentions of tourists in the P2P accommodation sector in developing nations have received scant empirical attention. Even in some developed countries, the majority of studies that have been published in the literature to date have not offered a thorough analysis of the causes of behavioral intentions in the P2P accommodation sector. Therefore, by undertaking a quantitative analysis of the antecedents of guests’ behavioral intentions in Malaysia, this study may contribute to the literature on services marketing and the P2P accommodation industry. The current study may be able to offer a reliable source of data on the physical environment, social environment, feelings, satisfaction, and behavioral intentions of guests in the P2P accommodation sector and present potential for conducting more research in this area.

Previous research (Tussyadiah, 2016; Guttentag et al., 2018) generally concentrated on antecedents such as household amenities, pricing, authenticity, social benefits, and economic benefits, but the social environment was excluded as an antecedent. Nine guests who had stayed in Malaysia’s P2P accommodation sector through Airbnb were questioned by Razli et al. (2017). They discovered that majority of guests stayed at a P2P accommodation in Malaysia because of its lower cost. However, the study’s primary focus was on Airbnb guests. They did not look at the patrons of homestays, which account for a sizable portion of Malaysia’s P2P accommodation sector.

The study’s findings will aid in a better understanding of the Mehrabian and Russell (1974) S-O-R model, which emphasizes the social environment as a stimulus. The external stimuli, guest satisfaction, and guest emotions that correlate to the internal states and behavioral intentions of the guest as response are denoted in this study by the physical and social environments related to P2P accommodations. The elements of the P2P experience that influence guests’ behavioral intentions by utilizing both internal states and external stimuli were established by this study. As mentioned, the antecedent social environment drives the guest’s emotions and is thought to be important while they are staying at a P2P accommodation.

In terms of P2P accommodations, not many studies have focused on the guests’ behavioral intentions. Therefore, it has been suggested that future research focusing on P2P accommodations must give the foundation that permits establishing other environmental factors that could boost visitor loyalty and satisfaction. The study’s findings were in line with academic works that had scientifically investigated how the physical environment affected satisfaction, emotions, and behavioral intentions (Bitner, 1992; Wakefield and Blodgett, 1996; Ali et al., 2016; Moon et al., 2016). Additionally, these results are in line with past research on the social environment (Ali and Omar, 2014; Tussyadiah, 2016; Stienmetz et al., 2020; Wang et al., 2021).

The research on P2P hosts emphasizes the need of providing a welcoming environment and recommending nearby events, experiences, and activities. The research on P2P accommodation services, like Airbnb and homestays, shows how important it is to educate hosts (especially those who share a home with the guests) on hospitality standards, different ways to actively engage guests in local experiences, and efficient pricing tactics. The findings also recommend P2P platforms to use market segmentation tactics rather than repeatedly telling the same narrative about their products. Additionally, P2P websites can enhance their marketing campaigns by incorporating private and homelike elements relevant to the property to influence visitor behavior. The results suggest that hoteliers can showcase their own offerings through various programs and build brands by fusing local and social experiences to compete with P2P accommodation in terms of social experiences.

This research has a number of theoretical implications that can be drawn. By identifying many antecedents that influence guests’ behavioral intentions, this study expanded earlier research on P2P accommodations and consumer behavior. Results from this study also offered definitive evidence of the efficacy of these behavioral driving forces. The results also offered meaningful support for the idea that P2P accommodation is a practical application of the stimulus-organism-response theory, which postulates that behavioral intentions are influenced by mutual benefits and commercial sharing systems. This study also revealed one of the key characteristics of the P2P experience: guests’ behavioral intentions were influenced by both internal and external inducements. Particularly, excellent ambiance, design, spatial layout, and social setting satisfied guests and boosted their visitation. The physical environment also inspired visitors, aroused feelings, and generated behavioral intentions. This proves the theory that P2P accommodations with high-quality amenities at lower prices encourage more return visits. By creating the construct behavioral intention based on a hypothetical model through the lens of the stimulus-organism-response theory, this study also provided a broader perspective on the actions of visitors. The study’s findings supported earlier literary models (Mehrabian and Russell, 1974; Jang and Namkung, 2009; Ali and Amin, 2014).

The findings also have practical implications for P2P accommodation platforms like Airbnb and homestays that aim to educate hosts on hospitality standards, amenities, efficient pricing strategies, and ways to engage guests in local experiences. Additionally, it should be recognized that interactions with hosts and other guests have little impact on how guests feel. This suggests that guests’ experiences rely on the kinds of rooms they book at P2P accommodation. There are two distinct groups in the market, and each has a different attitude toward social practices. In comparison to those who rent a complete home or apartment, guests who stay with hosts in the same room require more social interactions. Due to the possibility that the guests who choose to stay with the host are more socially inclined than other guests, P2P platforms must therefore adopt market segmentation strategies rather than concentrating on communications.

## Limitations and suggestions for future studies

This study, like all others, has some limitations on what can be explored in the future. The only sources of information used to compile the data for this study were online survey questionnaires; no in-person interviews were conducted. Interviewees may be more comfortable sharing intimate details and experiences in person. Additionally, if the researchers are unclear, they will be able to seek for clarification from the interviewees. Therefore, it is advised that future studies conduct in-person interviews with guests staying at P2P accommodations in Malaysia.

Next, the study limited its attention to a few potential predictors of guests’ behavioral intents to book P2P accommodations. Only a portion of the diversity in guests’ behavioral intentions could be explained by the study’s antecedents. Other variables that were left out of this study could have a bigger impact on guests’ behavioral intentions when staying in P2P accommodations. Additional study should include more components in the research model for studies of consumer behavior, such as location benefits, local food, communication channels and innovation.

Further, future research must further test and validate the model in various contexts since this study involved both local and foreign tourists who had stayed in Malaysia. Future research can also make arguments based on the distinctions between users of other P2P accommodation platforms and homestays, as the study focused on homestays in Malaysia. The distinctions that might exist between guests who share space with the hosts and those who do not share space should be examined to support this study on the importance of social environment. Future research should examine elements that may affect hotel experiences and influence consumers’ decisions to use P2P.

## Data availability statement

The raw data supporting the conclusions of this article will be made available by the authors, without undue reservation.

## Ethics statement

The studies involving human participants were reviewed and approved by Universiti Putra Malaysia. The participants provided their written informed consent to participate in this study.

## Author contributions

LA, FYW, and SIN contributed to conception and design of the study. LA wrote the first draft of the manuscript. YY, FYW, SIN, and X-JL contributed to improve and revise the manuscript revision. All authors contributed to the article and approved the submitted version.

## Funding

This research was supported by the Universiti Putra Malaysia Putra Grant (Project Code: GP/2017/9563500).

## Conflict of interest

The authors declare that the research was conducted in the absence of any commercial or financial relationships that could be construed as a potential conflict of interest.

## Publisher’s note

All claims expressed in this article are solely those of the authors and do not necessarily represent those of their affiliated organizations, or those of the publisher, the editors and the reviewers. Any product that may be evaluated in this article, or claim that may be made by its manufacturer, is not guaranteed or endorsed by the publisher.
